# Anti-VEGF treatment: a personal perspective from Rwanda

**Published:** 2025-01-31

**Authors:** Michael Mikhail

**Affiliations:** 1 Consultant Ophthalmologist and Vitreoretinal Surgeon: Kabgayi Eye Unit, Muhanga, Rwanda and Senior Lecturer: College of Medicine and Pharmacy, University of Rwanda.

**Despite insurance covering much of the cost of treatment, affordability can still be a barrier, especially for patients who live in remote areas.**

**Figure:**
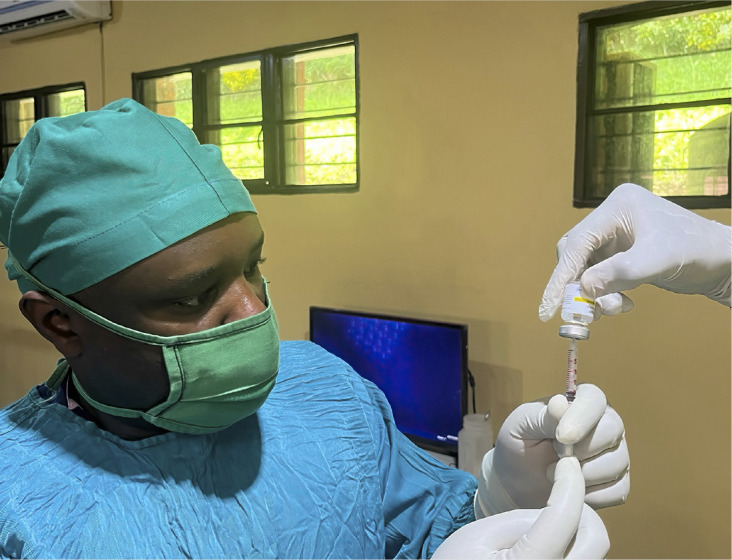
Preparing an anti-VEGF injection. RWANDA

© MICHAEL MIKHAIL CC BY-NC-SA 4.0

Kabgayi Eye Unit (KEU) is a tertiary referral centre for eye care based in the southern province of Rwanda. We also serve patients from neighbouring countries like Uganda, the Democratic Republic of Congo (DRC), and Burundi.

## Treatment landscape

Despite the advances in anti-VEGF agents, bevazucimab (Avastin) remains the mainstay in my practice for its proven efficacy and effectiveness^1^ and, more importantly, its affordability. We are fortunate in Rwanda, as we have a sustainable supply of bevacizumab from authorised Roche pharmaceutical dealers. As is the case in many countries, bevazucimab is not approved by the Rwandan Food and Drug Authority (FDA) for ocular conditions, and it's use is ‘off-label’. However, it was added to the World Health Organization Essential Medicines List for the treatment of neovascular (wet) age-related macular degeneration in 2013. The dose we use is 2.5 mg in 0.1 ml. 

## Patient access and affordability

The cost to the patient of a single bevacizumab injection is around 30,000 Rwandan Franc (equivalent to US $25). With over 90% of the population covered by health insurance, mainly through community-based schemes, the financial burden of treatment is significantly reduced. Patients typically only pay 10–15% of the treatment cost, with insurance covering the rest. Approval for reimbursement is required through the Rwanda Social Security Board (RSSB) before commencing treatment. This is done online by the KEU administration team with instant approval from RSSB, making treatment accessible to most patients without any delay.

## Adherence to treatment plan

For bevacizumab naïve patients, I recommend three doses, one every four weeks, followed by a pro re nata (PRN) or ‘as needed’ approach, based on disease activity. Adherence to the treatment plan is generally good, with most patients following through with scheduled injections. However, treatment gaps can be more than 4 weeks apart, depending on individual circumstances. I found that the number of injections and perceived lack of significant vision improvement may affect some patients’ adherence.

## Barriers to treatment

Even though insurance covers much of the cost in Rwanda, some people still struggle to afford treatment. For patients in remote areas, the cost of travel may be an issue. A patient's overall health can also affect how well they adhere to their treatment schedule and handle the injections. Finally, some patients doubt that the treatment is helping them, especially those with modest visual improvement.

## Our management protocol

We use an Avastin vial of 100 mg/4 ml, kept refrigerated in theatre between 2°C and 8°C. A single vial can treat 30–35 patients. We do not have a compounding pharmacy for pre-made sterile Avastin injections. It is well known that poor compounding can increase the risk of infection, and there is good evidence that multiple direct withdrawal from the vial is safe.^2^ Our experience at KEU agrees with this. We record the batch number of each vial in a register before opening it, with the date of opening written on the bottle. We don't have a specific time frame for using an Avastin vial, but as we are a high-volume centre, we usually finish it within a week. We do all Avastin intravitreal injections in the operating theatre; the injection is given by an ophthalmologist trained in this procedure. Withdrawal and preparation of the medication is usually done by the scrub nurse/assistant.

For injection technique, consult this article: www.cehjournal.org/articles/754. This is similar to our injection technique here at KEU.

Endophthalmitis is a dreaded complication of intraocular surgeries, leading to severe ocular morbidity and vision/eye loss. Therefore, meticulous preparation of the drug and sterile injection technique cannot be stressed enough, especially when multiple injections from the same vial are used, as this can increase the risk of cluster endophthalmitis (where multiple cases of endophthalmitis can occur in close proximity to each other).

Over the last 5 years (2019–2023) we performed 3,383 injections of Avastin with only two cases of endophthalmitis during the same time.

## References

[1] Tors S., Anderson S. (2023). How do anti-vascular endothelial growth factors (anti-VEGFs) compare with one another for people with diabetic macular edema?. Cochrane Clinical Answers..

[2] Das T., Volety S., Ahsan SM. (2015). Safety, sterility and stability of direct-from-vial multiple dosing intravitreal injection of bevacizumab.. Clin Exp Ophthalmol..

